# Differential Serotonin Uptake Mechanisms at the Human Maternal–Fetal Interface

**DOI:** 10.3390/ijms22157807

**Published:** 2021-07-21

**Authors:** Petra Baković, Maja Kesić, Maja Perić, Ivona Bečeheli, Marina Horvatiček, Meekha George, Lipa Čičin-Šain, Gernot Desoye, Christian Wadsack, Ute Panzenboeck, Jasminka Štefulj

**Affiliations:** 1 Department of Molecular Biology, Ruđer Bošković Institute, Bijenička cesta 54, HR-10000 Zagreb, Croatia; petra.bakovic@irb.hr (P.B.); maja.kesic@irb.hr (M.K.); maja.peric@irb.hr (M.P.); ivona.beceheli@irb.hr (I.B.); marina.horvaticek@irb.hr (M.H.); lipa.cicin-sain@irb.hr (L.Č.-Š.); 2 Otto Loewi Research Center, Division of Immunology and Pathophysiology, Medical University of Graz, Heinrichstrasse 31a, A-8010 Graz, Austria; meekha.george@medunigraz.at; 3 Department of Obstetrics and Gynaecology, Medical University of Graz, Auenbruggerplatz 14, A-8036 Graz, Austria; gernot.desoye@medunigraz.at (G.D.); christian.wadsack@medunigraz.at (C.W.)

**Keywords:** 5-HT, placenta, cord blood, SERT, PMAT, organic cation transporters, trophoblasts, feto-placental endothelial cells, platelets

## Abstract

Serotonin (5-HT) plays an extensive role during pregnancy in regulating both the placental physiology and embryonic/fetal development. The uptake of 5-HT into cells is central to the control of local concentrations of 5-HT near its molecular targets. Here, we investigated the mechanisms of 5-HT uptake into human primary placental cells and cord blood platelets, all isolated immediately after birth. Trophoblasts and cord blood platelets showed 5-HT uptake with similar Michaelis constant (*Km*) values (~0.6 μM), typical of the high-affinity serotonin transporter (SERT). The uptake of 5-HT into trophoblasts was efficiently inhibited by various SERT-targeting drugs. In contrast, the uptake of 5-HT into feto-placental endothelial cells was not inhibited by a SERT blocker and showed a *Km* value (~782 μM) in the low-affinity range. Consistent with this, *SERT* mRNAs were abundant in term trophoblasts but sparse in feto-placental endothelial cells, whereas the opposite was found for the low-affinity plasma membrane monoamine transporter (*PMAT*) mRNAs. Organic cation transporter (*OCT*) *1*, *2*, and *3* mRNAs were absent or sparse in both cell types. In summary, the results demonstrate, for the first time, the presence of functional 5-HT uptake systems in feto-placental endothelial cells and fetal platelets, cells that are in direct contact with fetal blood plasma. The data also highlight the sensitivity to various psychotropic drugs of 5-HT transport into trophoblasts facing the maternal blood. The multiple, high-, and low-affinity systems present for the cellular uptake of 5-HT underscore the importance of 5-HT homeostasis at the maternal–fetal interface.

## 1. Introduction

Serotonin (5-hydroxytryptamine, 5-HT) is a multifunctional bioamine, best known as a brain neurotransmitter involved in mood, sleep and appetite regulation, as well as in various neuropsychiatric disorders. In addition, 5-HT acts as an endocrine, paracrine or autocrine agent regulating various peripheral functions such as vascular tone, hemostasis, intestinal motility, immune response, bone remodeling and the energy metabolism [1,2]. During pregnancy, 5-HT mediates the fine-tuning of embryonic/fetal development [3] and plays an important role in placental physiology [4]. It modulates a number of cellular processes in placental cells, including proliferation, cell viability, cell cycle progression, apoptosis, and estrogen production [5,6,7,8,9]. As a potent vasoactive autacoid, 5-HT also influences the utero-placental and feto-placental blood flow [10,11]. Early in pregnancy, the placenta supplies the developing fetus with maternal and/or placenta-derived 5-HT, which is required for proper fetal (neuro)development [12,13,14]. This wide range of functions may account for the findings linking altered placental 5-HT homeostasis to pregnancy disorders, such as preeclampsia [15], fetal growth restriction [16], and gestational diabetes mellitus [17], as well as to mental health implications in the offspring [18,19,20,21,22].

5-HT exerts its physiological effects by interacting with 14 different plasma membrane-bound 5-HT receptors coupled to various intracellular signaling pathways [23]. In addition, it regulates some physiological functions in a receptor-independent manner, by directly binding to some extracellular and cytosolic proteins [24]. Recent evidence showed that 5-HT also binds to histone proteins and is, thus, involved in transcriptional regulation [25]. All these effects depend on the availability of 5-HT in close proximity to its molecular targets. Systems for the transport of 5-HT across biological membranes efficiently control local 5-HT concentrations and, thus, play a central role in governing 5-HT actions.

There are two distinct plasma membrane transport systems for 5-HT, uptake-1 and uptake-2, characterized by high-affinity/low-capacity and low-affinity/high-capacity kinetic properties, respectively. The uptake-1 system for 5-HT is represented by the serotonin transporter (SERT), a highly specific high-affinity 5-HT carrier [26]. SERT is best known for mediating the reuptake of 5-HT in the brain but is also expressed in many peripheral tissues, including the placenta [27].

Due to its central role in the clearance of 5-HT from the synaptic cleft, SERT is an important target of many psychotropic drugs, such as tricyclic antidepressants (TCAs) and selective serotonin reuptake inhibitors (SSRIs). In adults, SERT also represents a major mechanism responsible for regulating 5-HT concentrations in blood plasma by mediating the uptake of 5-HT into platelets [28]. However, an analogous 5-HT uptake mechanism in fetal circulation has not yet been described.

In contrast to SERT, carriers of the uptake-2 system, including the plasma membrane monoamine transporter (PMAT) and the organic cation transporters 1 (OCT1), 2 (OCT2), and 3 (OCT3), exhibit low substrate affinity and readily transport various organic cations [29]. In addition to being involved in the regulation of neurotransmitters and other endogenous compounds, these polyspecific carriers also play an important role in the distribution and elimination of drugs and other xenobiotics. Uptake-2 transporters show widespread and overlapping expression in many organs, including the placenta. In human term placenta, *OCT3* is predominantly expressed, while *OCT1*, *OCT2*, and *PMAT* are expressed at very low levels [30,31].

The placenta is a complex, heterocellular organ interposed between the maternal and fetal blood. Among others, two main cell types that form the maternal–fetal blood barrier are trophoblasts covering the surface of the villous trees and facing the maternal blood, and feto-placental endothelial cells, which form the feto-placental vessels and are in direct contact with the fetal blood. There is evidence for the placental uptake of 5-HT from both maternal and fetal circulation at the end of human pregnancy.

High-affinity uptake of 5-HT from maternal blood was first suggested by transport studies of 5-HT in plasma membrane vesicles isolated from human term placenta [27]. An analogous high-affinity uptake system for 5-HT, sensitive to TCAs and SSRIs, has been described in the human trophoblast-like choriocarcinoma cell line JAR [32]; however, to our knowledge, has not been demonstrated in primary trophoblasts. Regarding placental uptake of 5-HT from fetal blood, a low-affinity 5-HT uptake mechanism mediated by OCT3 present in the basal membrane was recently proposed by 5-HT transport studies in plasma membrane vesicles of human term placenta and by an in situ dual perfusion system in rat term placenta [33].

Despite the growing interest in the feto-placental 5-HT system and various expression studies, the kinetic and pharmacological properties of 5-HT uptake in primary trophoblasts have not been investigated. There is also a clear lack of functional studies of possible 5-HT uptake into cells in direct contact with fetal plasma, such as feto-placental endothelial cells and fetal platelets. We hypothesized that these cells have functional systems for the active uptake of 5-HT. This is important because such systems will contribute to the regulation of fetal circulating 5-HT levels. To better understand the mechanisms that regulate 5-HT homeostasis during human fetal development, we examined and characterized 5-HT uptake in primary trophoblasts, feto-placental endothelial cells, and cord blood platelets, all isolated from human tissues immediately after birth.

## 2. Results

### 2.1. 5-HT Uptake into Primary Placental Cells

**Figure 1:**
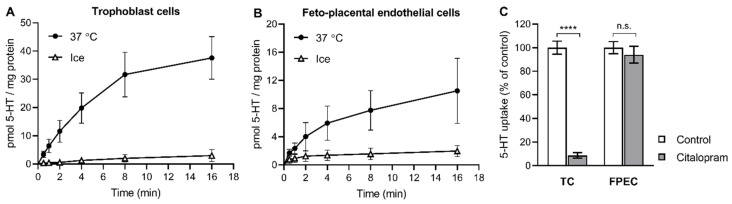
(**A**,**B**) Time- and temperature-dependent uptake of 5-HT into primary placental cells. Human primary (**A**) trophoblasts and (**B**) feto-placental endothelial cells were incubated in the presence of radiolabeled 5-HT (10^−7^ M) at 37 °C or on ice for 0.5 to 16 min. Values are the means ± SEM from two donors, each analyzed in triplicate. (**C**) The effect of citalopram on the uptake of 5-HT into primary placental cells. Trophoblast cells (TC) and feto-placental endothelial cells (FPEC) were incubated for 10 min in the presence of radiolabeled 5-HT (10^−7^ M) and citalopram (10^−7^ M) or vehicle (control). The specific uptake was calculated as the difference between transport at 37 °C and on ice and expressed as a percentage of the control. Values are the means ± SEM of two independent experiments, each performed in two or three replicates. ^****^ *p* < 0.0001 compared to vehicle, n.s. not significant (Sidak’s multiple comparisons test following two-way ANOVA).

We first examined whether 5-HT was taken up by cells of both sides of the human placental barrier. Human primary trophoblasts (Figure 1A) and feto-placental endothelial cells (Figure 1B) both showed time- and temperature-dependent accumulation of radiolabeled 5-HT (0.1 μM), indicating the presence of a specific carrier-mediated transport mechanism (Figure S1). Specific 5-HT uptake into trophoblasts was efficiently inhibited by citalopram (Figure 1C), a potent and highly selective blocker of SERT [34]. This clearly demonstrates a role of the high-affinity 5-HT transporter in trophoblasts. In contrast, the specific 5-HT uptake into feto-placental endothelial cells was not affected by citalopram (Figure 1C), indicating the absence of a functional SERT-mediated uptake system in feto-placental endothelial cells.

**Figure 2:**
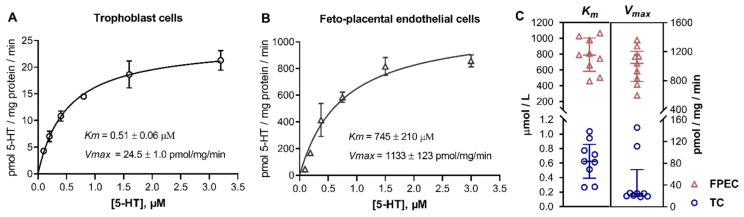
Kinetic analysis of the uptake of 5-HT into human primary placental cells. Human primary (**A**) trophoblasts and (**B**) feto-placental endothelial cells were incubated in the presence of six increasing ^3^H-5-HT concentrations between (**A**) 0.1 and 3.2 μM and (**B**) 94 and 3000 μM for 2 and 8 min, respectively. The specific uptake was calculated as the difference between transport at 37 °C and on ice. The initial rates of specific uptake were plotted against substrate concentration and fitted to the Michaelis–Menten kinetics model. Data (mean ± SD, n = 3) from one of the nine subjects studied are shown. (**C**) Best-fit values of the Michaelis affinity constant (*Km*) and maximal transport velocity (*Vmax*) obtained in trophoblast cells (TC) and feto-placental endothelial cells (FPEC) isolated from different placentas (n = 9 for each cell type). Lines represent the medians and interquartile range (for numerical results, see Table S1). Values of *Km* and *Vmax* were significantly different between trophoblasts and feto-placental endothelial cells (*p* < 0.0001 in both cases; *t*-test or Mann–Whitney test, as appropriate).

Next, we performed kinetics studies in which we measured the initial rates of specific 5-HT uptake across multiple substrate concentrations covering a range typical of high-affinity (uptake-1) and low-affinity (uptake-2) transport systems. As expected, trophoblasts (Figure 2A) showed saturation curves over the high-affinity range of 5-HT concentrations (0.1 to 3.2 μM), typical of SERT-mediated uptake. In contrast, 5-HT uptake into feto-placental endothelial cells was not saturable over the high-affinity range of 5-HT concentrations (Figure S2) but exhibited saturation kinetics compliant with the Michaelis–Menten model only over the low-affinity range of 5-HT concentrations (94 to 3000 μM; Figure 2B).

To gain insight into inter-individual variability of kinetic parameters, we performed kinetics analyzes in multiple (nine for each cell type) donors. Figure 2C shows the calculated best-fit values of the Michaelis affinity constant (*Km*) and maximal transport velocity (*Vmax*). In all subjects, the *Km* values (mean ± SD, n = 9) in trophoblasts (0.64 ± 0.27 μM) were characteristic of high-affinity [26], and, in feto-placental endothelial cells (782 ± 218 μM), of low-affinity [35,36,37,38,39,40] transport systems.

### 2.2. Expression of 5-HT-Regulating Genes in Primary Placental Cells

**Figure 3:**
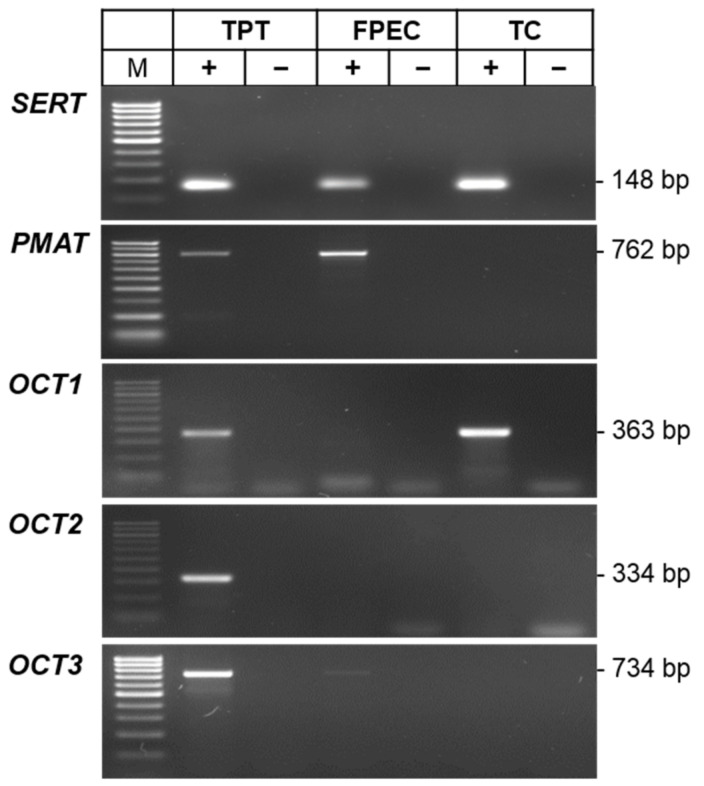
Conventional end-point RT-PCR analysis of serotonin transporter (*SERT*), plasma membrane monoamine transporter (*PMAT*), organic cation 1 (*OCT1*), 2 (*OCT2*), and 3 (*OCT3*) mRNAs in pooled samples of human primary trophoblasts (TC; n = 9) and feto-placental endothelial cells (FPEC; n = 12). The total placental tissue (TPT; a pool from 20 individuals) served as a positive control. Reactions were prepared with (+) or without (−) addition of reverse transcriptase in the cDNA synthesis step. The size of specific amplicons in base pairs (bp) is indicated. M, 100 bp molecular standard.

To substantiate our functional findings and identify the molecular players responsible for the different 5-HT uptake kinetics in trophoblasts and feto-placental endothelial cells, we analyzed the mRNA expression of uptake-1 and -2 transporters. First, we examined the presence of *SERT*, *PMAT*, *OCT1*, *OCT2*, and *OCT3* mRNAs in pooled samples of trophoblast cells (n = 9) and feto-placental endothelial cells (n = 12), using the total placental tissue (pool of 20 samples) as a positive control. Qualitative end-point RT-PCR detected *SERT* mRNAs in both trophoblasts and feto-placental endothelial cells, *PMAT* and very weak *OCT3* signals only in fetoplacental endothelial cells, and *OCT1* signals only in trophoblasts, while *OCT2* signals were below the detection limit in both cell types (Figure 3). More sensitive real-time qPCR analysis (Figure S3) confirmed the *OCT2* and *OCT3* findings but revealed the presence of *PMAT* and *OCT1* signals also in trophoblasts and feto-placental endothelial cells, respectively, albeit at very low levels, as indicated by the late quantification cycle (Cq) values.

**Figure 4:**
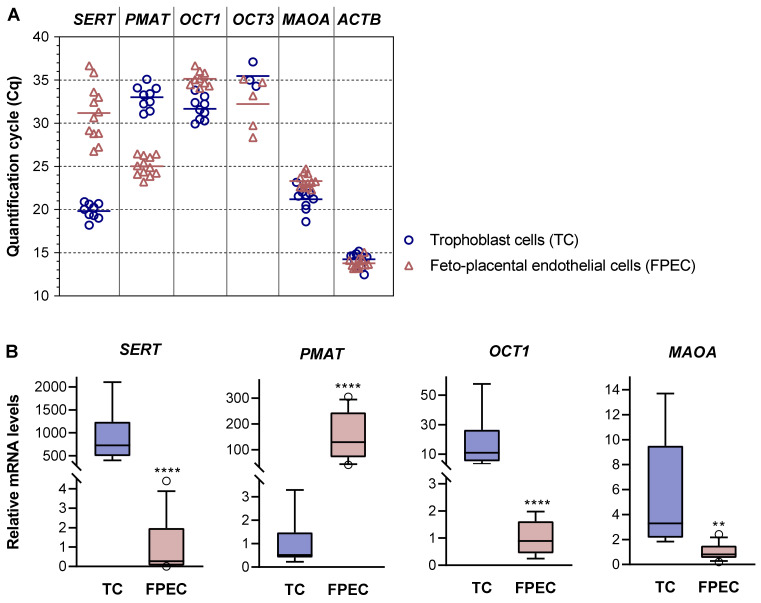
Expression of 5-HT regulating genes in primary placental cells. (**A**) Quantification cycle (Cq) values obtained by RT-qPCR analysis of serotonin transporter (*SERT*), plasma membrane monoamine transporter (*PMAT*), organic cation 1 (*OCT1*), organic cation 3 (*OCT3*), monoamine oxidase A (*MAOA*), and actin beta (*ACTB*) mRNAs in human primary trophoblasts (n = 9) and feto-placental endothelial cells (n = 12). A specific *OCT1* signal was detected in 9 of the 12 feto-placental endothelial cell samples analyzed. A specific *OCT2* signal was absent in all trophoblast and feto-placental endothelial cell isolations. A specific *OCT3* signal was detected in 3 of 9 trophoblast and 5 of 12 feto-placental endothelial cell samples. For numerical results, see Table S2. (**B**) Relative expression levels of *SERT*, *PMAT*, *OCT1*, and *MAOA* mRNAs normalized to *ACTB* mRNA. For each gene, the mean of the cells with the lower expression level was arbitrarily set to 1.00. Data are presented as boxplots with whiskers indicating the 10th and 90th percentiles and circles showing individual values outside the whisker range (n = 9 for TC; n = 12 for FPEC, except for *OCT1* where n = 9). ^**^ *p* < 0.01, ^****^ *p* < 0.0001 (*t*-test or Mann–Whitney test, as appropriate).

Next, we performed real-time qPCR analysis in individual samples of trophoblasts and feto-placental endothelial cells isolated from different donors (Figure 4 and Table S2). Consistent with the results of 5-HT uptake studies, all trophoblast samples were rich in *SERT* mRNA, as indicated by low Cq values (Figure 4A). In contrast, feto-placental endothelial cells yielded generally high (mean 31.2) and largely variable (range 26.8 to 36.7) Cq values. The relative levels of *SERT* mRNAs were approximately 1000-fold lower in feto-placental endothelial cells than in trophoblasts (*p* < 0.0001) and differed by up to 400-fold between feto-placental endothelial cells isolated from different placentas (Figure 4B, first graph). This is consistent with previous immunohistochemical studies that reported weak staining for the SERT occasionally observed on the feto-placental endothelium of human term placentas [14,41]. Combined with our 5-HT uptake results, the findings of sporadic expression of low *SERT* levels suggest that this carrier is not essential in feto-placental endothelial cells.

In contrast to *SERT*, *PMAT* mRNAs were abundant in feto-placental endothelial cells but rare in trophoblasts (Figure 4A). The relative levels of *PMAT* mRNAs were approximately 300-fold higher in feto-placental endothelial cells compared with in trophoblasts (*p* < 0.0001; Figure 4B, second graph).

*OCT1* transcripts, found at low levels in total placental tissue (Cq 31.4), were detected in all nine trophoblast samples and in 9 of 12 feto-placental endothelial cell samples, but at rather low/negligible levels (Figure 4A). *OCT2* mRNAs, detected at low levels in total placental tissue (Cq 30.5), were absent in all cell samples analyzed. *OCT3* mRNAs, which were abundant in total placental tissue (Cq 22.0), were detected at very low levels in 3 of 9 trophoblast samples and in 5 of 12 feto-placental endothelial cell samples (Figure 4A) but were absent in the majority of samples.

Our results on *OCT3* expression are consistent with reports on the absence of OCT3 protein in microvillous tissue [42] and syncytiotrophoblast [14] of human term placentas, as well as on the absence of *OCT3* mRNA in the human trophoblast cell lines BeWo, JEG-3 [31], and JAR [43]. They may reflect the presence of OCT3 in endothelial cells from smaller vessels [44], in other populations of trophoblasts, or in other types of placental cells, such as fibroblasts or macrophages. The localization of OCT1, OCT2, and PMAT in the placenta has not yet been analyzed (reviewed in [45]).

Taken together, the negligible or absent expression of uptake-2 transporters suggests a central role for high-affinity, SERT-mediated mechanism of 5-HT uptake in villous trophoblasts. The data also suggest that PMAT is the central transporter responsible for low-affinity 5-HT uptake into feto-placental endothelial cells. We additionally analyzed the expression of monoamine oxidase (MAOA), a rate-limiting enzyme in the catabolism of 5-HT [46]. *MAOA* mRNAs were abundant in both cell types, with 4.9-fold higher levels (*p* < 0.01) in trophoblasts compared with in feto-placental endothelial cells (Figure 4B, fourth graph).

### 2.3. Uptake of 5HT in Cord Blood Platelets

**Figure 5:**
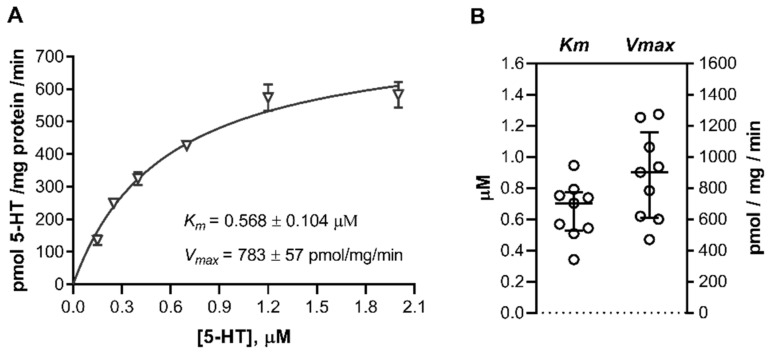
Uptake of 5-HT into human cord blood platelets. Platelets were isolated from cord blood samples and incubated for 1 min in the presence of six increasing concentrations (0.15 to 2.00 μM) of radiolabeled 5-HT. The specific transport was determined as the difference between transport at 37 °C and at approximately 4 °C (ice bath). (**A**) Initial rates of specific uptake of 5-HT were plotted against the substrate concentration and fitted to the Michaelis–Menten kinetics model. Shown are data (means ± SD) from one of nine subjects studied. (**B**) Best-fit values of the Michaelis affinity constant (*Km*) and maximal transport velocity (*Vmax*). Circles show individual values obtained in cord blood platelets isolated from different subjects (n = 9); lines represent medians and interquartile range. For numerical data, see Table S1.

To investigate possible additional mechanisms contributing to uptake of 5-HT from the fetal circulation, we examined the transport of 5-HT into platelets isolated from cord blood samples. Cord blood platelets showed efficient, time-, and temperature-dependent 5-HT uptake, with initial rates of specific 5-HT transport saturating over the high-affinity range of 5-HT concentrations (0.15 to 2.0 μM; Figure 5A). *Km* values (0.65 ± 0.18 μM, n = 9; Figure 5B) were typical of high-affinity uptake [26] in all subjects tested and similar to those found in trophoblasts (Figure 2C and Supplementary Table S1) and previously in adult platelets [47,48].

The *Vmax* values expressed per platelet count were also comparable between platelets from cord blood (109 ± 35 pmol/10^8^ platelets/min) and adult platelets (142 ± 25 pmol/10^8^ platelets/min) [47,48]. Expressed per total cellular protein, *Vmax* values were on average 20-fold higher (*p* < 0.0001, Mann–Whitney test) in cord blood platelets (Figure 5B) than in trophoblasts (Figure 2C; see also Table S1). This is consistent with the fact that platelets are among the few cell types with the highest amount of SERT protein in the adult human body [49].

### 2.4. Effects of Various Psychotropic Drugs on the Uptake of 5-HT in Human Primary Trophoblasts

**Figure 6:**
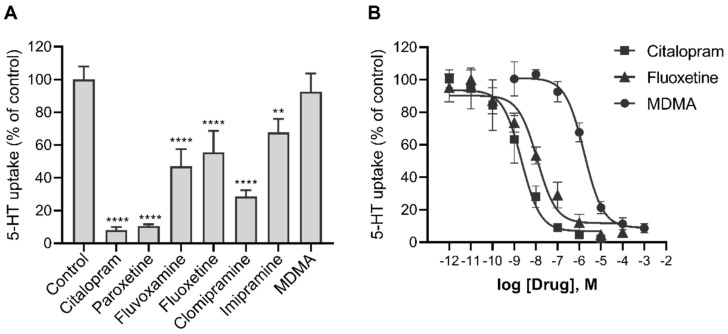
The potency of various psychotropic drugs to inhibit the uptake of 5-HT into human primary trophoblasts. Cells were incubated for 10 min in the presence of radiolabeled 5-HT (10^−7^ M) and (**A**) 10^−7^ M or (**B**) increasing the concentrations of the indicated drugs. Values are expressed as a percentage of control (vehicle without the drug). The best-fit half-maximal inhibitory concentrations (IC_50_) of citalopram, fluoxetine, and MDMA were 2, 11, and 1678 nM, respectively, with 95% confidence intervals (CI) of 1 to 7 nM, 4 to 33 nM and 1045 to 2745 nM, respectively. All data are the means ± SEM of three separate experiments, each performed in triplicate. Cells from different donors were used to generate the data shown in panels A and B; the values for fluoxetine in panel A (44%) are not statistically different from the values for 10^−7^ M fluoxetine in panel B (29%). ^**^ *p* < 0.01, ^****^ *p* < 0.0001 for comparison with control (Dunnett’s test after one-way ANOVA, F_7,16_ = 52.34, *p* < 0.0001). MDMA—3,4-methylenedioxy-methamphetamine.

Since trophoblasts are a primary cell type on which all drugs administered to the mother would act in the human feto-placental unit, we additionally tested the potency of some of the widely used SERT-targeting antidepressants to inhibit the uptake of 5-HT into human primary trophoblasts. The uptake of 5-HT into trophoblasts was decreased in the presence of submicromolar (10^−7^ M) concentrations of all antidepressants tested (Figure 6A), namely citalopram (by 92%), paroxetine (by 89%), fluoxetine (by 44%), fluvoxamine (by 53%), clomipramine (by 71%), and imipramine (by 32%).

The psychoactive stimulant 3,4-methylenedioxy-methamphetamine (MDMA, “Ecstasy”), which interacts with SERT, also inhibited the transport of 5-HT into trophoblasts, but with a lower potency. Specifically, MDMA showed an IC_50_ value in the range of 10^−6^ M, while those of citalopram and fluoxetine were only in the range of 10^−9^ and 10^−8^ M, respectively (Figure 6B).

## 3. Discussion

In the present study, we investigated 5-HT uptake mechanisms in human primary placental cells and cord blood platelets, all isolated immediately after birth. To determine the uptake kinetics, we performed extensive initial rate studies by radiotracer assay. The initial rates of specific 5-HT uptake, calculated as the difference between the total uptake (at 37 °C) and nonspecific uptake (at approximately 4 °C), were measured at multiple substrate concentrations, covering a range typical of both high-affinity (uptake-1) and low-affinity (uptake-2) transport systems. This allowed the estimation of the kinetic parameters *Km* (approximating the affinity of a carrier for 5-HT) and *Vmax* (describing the maximal uptake velocity reached when the carrier is fully saturated). In addition, we examined the mRNA levels of 5-HT carriers in primary placental cells and investigated the pharmacological properties of 5-HT uptake into primary trophoblasts.

Our results show that human primary trophoblasts expressed *SERT* mRNA and took up 5-HT with the high-affinity transport kinetics characteristic of a SERT-mediated process. The *Vmax* values of 5-HT uptake in primary trophoblasts (18–146 pmol/mg/min, n = 9) were similar to those reported for plasma membrane vesicles isolated from human native placentas (25.6–270 pmol/mg/min) [27,50,51,52,53], but much higher than those reported for the trophoblast-like cell line JAR (0.88–1.58 pmol/mg/min) [32,53,54,55]. These findings suggest a loss of functional SERT protein in the commonly used JAR cells. They also indicate that primary human trophoblasts provide a credible physiological model for studying various regulatory features of placental 5-HT uptake from the maternal circulation in an intact cellular system.

Epidemiological data showed that SERT-targeting antidepressants are increasingly used in pregnancy [56,57]. Therefore, we investigated the ability of these drugs to affect the uptake of 5-HT into human primary trophoblasts, a primary cell type on which any drug administered to the mother would act in the human feto-placental unit. We found that the uptake of 5-HT into human primary trophoblasts was inhibited by nanomolar concentrations of common tricyclic (imipramine and clomipramine) and SSRI (citalopram, paroxetine, fluoxetine, and fluvoxamine) antidepressants, with citalopram and paroxetine showing the strongest inhibitory effects. Moreover, the uptake of 5-HT into trophoblasts was antagonized by the SERT-interacting recreational psychostimulant MDMA, the use of which also appears to be increasing in pregnant women [58]. It has been shown that both antidepressants and MDMA can actively cross the placenta and affect the developing fetal organs [59,60,61]. Our results in human primary trophoblasts together with previous findings in human placental explants [14] and plasma membrane vesicles [27,50,51] suggest that the placenta itself, specifically the side facing the maternal blood, may be a target of these drugs. The possible downstream consequences of impaired placental 5-HT homeostasis induced by inhibition of 5-HT uptake in mother-facing trophoblast cells require further investigation.

**Figure 7:**
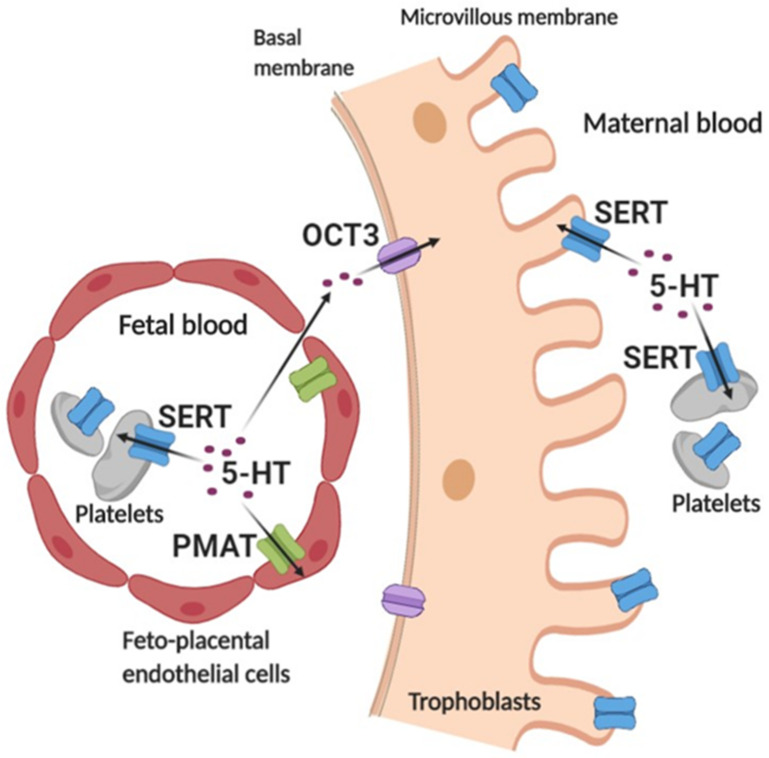
Schematic representation of the cellular uptake of serotonin from the placental extracellular space at the end of human pregnancy based on the literature and current study. Serotonin (5-HT) in fetal plasma is taken up into fetal platelets via the high-affinity serotonin transporter (SERT) and into feto-placental endothelial cells via the low-affinity plasma membrane monoamine transporter (PMAT). In addition, fetal 5-HT can diffuse via paracellular pathways between feto-placental endothelial cells and the placental stroma to the basal side of trophoblasts where it serves as a substrate of a low-affinity organic cation transporter 3 (OCT3) [33]. 5-HT in maternal plasma is sequestered via SERT, which is found on both maternal platelets and the apical side of trophoblasts. Created with BioRender.com.

The mechanisms involved in the uptake of 5-HT from the fetal circulation have only recently gained attention [33]. Here, we demonstrated, for the first time, a functional 5-HT uptake system at the end of human pregnancy in two cell types that are in direct contact with fetal blood plasma (Figure 7). First, we uncovered a low-affinity 5-HT uptake activity in feto-placental endothelial cells, most likely mediated predominantly by PMAT. The *Vmax* values of 5-HT uptake in feto-placental endothelial cells (1005 ± 251 pmol/mg/min) were lower than those originally reported for *PMAT*-transfected cells (6500 ± 200 pmol/mg/min) [62], but similar to those in human brain vascular smooth muscle cells (1034 ± 99 pmol/mg/min) [36] and higher than those in the human astrocytoma cell line 1321N1 (134 ± 16 pmol/mg/min) [37].

Second, we detected high-affinity 5-HT uptake activity in platelets isolated from cord blood, with kinetic parameters similar to those of SERT-mediated 5-HT transport in adult platelets [47,48]. We also detected the expression of mRNAs encoding MAOA, a 5-HT catabolizing enzyme with a high affinity for 5-HT, in feto-placental endothelial cells. This suggests that feto-placental endothelial cells can catabolize 5-HT to an inactive metabolite. In platelets, 5-HT, which has been taken up, can either be sequestered in dense granules or catabolized by MAOB [63]. Taken together, these findings suggest that both feto-placental endothelial cells and fetal platelets have systems in place to actively participate in the uptake and deactivation of 5-HT from the fetal circulation.

The observed *Km* values of 5-HT uptake in feto-placental endothelial cells (782 ± 218 μM) were approximately three orders of magnitude higher than those in cord blood platelets (0.65 ± 018 μM), suggesting that the two uptake systems operate at fundamentally different substrate concentrations. We speculate that feto-placental endothelial cells and fetal platelets work together to meet the requirements for maintaining optimal in vivo levels of extracellular 5-HT in different situations (Figure 7).

Under basal conditions, when fetal plasma 5-HT concentrations are in the low nanomolar range [64], the uptake of 5-HT is mediated mainly by the high-affinity system of fetal platelets. When fetal plasma 5-HT concentrations increase to levels at which the high-affinity system of platelets is fully saturated, the low-affinity system of feto-placental endothelial cells, which is saturated at much higher concentrations, contributes to bringing extracellular 5-HT to low basal levels. Another pathway of 5-HT sequestration in the human placenta, which functions especially at high 5-HT concentrations in fetal plasma, might involve the diffusion of 5-HT via paracellular pathways between feto-placental endothelial cells and the placental stroma to reach the basal membrane of the syncytium. There, it could serve as a substrate of the low-affinity OCT3-mediated uptake system [33].

Extracellular 5-HT concentrations may locally/transiently fluctuate to high levels as a consequence of 5-HT release from fetal platelets or other cells for endocrine, paracrine, or autocrine signaling purposes. Other situations in which the low-affinity 5-HT uptake systems present in feto-placental endothelial cells and the basal membrane may be important are when high-affinity SERT-mediated 5-HT uptake into fetal platelets is pharmacologically inhibited or impaired for genetic or pathological reasons. Indeed, maternal treatment with SSRI antidepressants has been associated with decreased 5-HT levels in cord blood platelets [65], suggesting inhibited uptake of 5-HT into fetal platelets. There is evidence that the 5-HT clearance system in the brain also combines high-affinity and low-affinity transporters [66]. The interplay between the different 5-HT uptake systems in the feto-placental unit should be further investigated.

We must acknowledge certain weaknesses of our study. Due to technical limitations, the different cell types studied were not obtained from the same individual or placenta, which precluded a direct comparison of cells from the same individual. To address this issue, we used a larger number of different donors for kinetic and gene expression analyzes. No pharmacological or knock-down studies have been performed to further support the notion of organic cation transporters 1 and 3 not contributing to low-affinity uptake of 5-HT into feto-placental endothelial cells. Primary placental cells were maintained in ambient atmosphere rather than under physiological oxygen conditions. Oxygen levels were previously reported to modulate the activity of SERT in some cellular models [67] and, thus, may have influenced the results. Using incubation on ice as measure of non-carrier-mediated uptake may have overestimated the carrier-mediated transport as passive diffusion is also affected by cooling. However, pharmacological experiments demonstrated a small diffusion, as almost complete uptake inhibition occurred at high inhibitor concentrations.

Trophoblasts isolated from different donors showed considerable inter-individual variability in *Vmax* (up to 8.1 fold) and *Km* (up to 3.9 fold) values. There was also considerable inter-individual variability in *Vmax* and *Km* values of feto-placental-endothelial cells (both up to 2.3 fold) and fetal platelets (up to 2.7 and 2.8 fold, respectively). This could be a consequence of the influence of various fetal and/or maternal factors, which were not controlled for in the present study. Indeed, fetal sex [33] and genotype [68] have been reported to influence the uptake of 5-HT into plasma membrane vesicles isolated from rat and human placentas, respectively. Several other gestational factors, such as maternal obesity and glucose tolerance [17,41,69,70] as well as maternal genotype, nutrition, stress, and immune activation [22], also influence the placental and fetal 5-HT homeostasis. Understanding the regulatory influences of various factors on the 5-HT uptake systems in the placenta and platelets, which is beyond the scope of the present study, requires further investigation with full kinetic measurements.

In summary, we comprehensively characterized the kinetic and pharmacological aspects of 5-HT uptake in primary trophoblasts facing maternal blood at the end of human pregnancy. In addition to establishing these cells as a valuable tool for studying various regulatory features of placental 5-HT uptake from the maternal circulation, the results highlight the sensitivity of placental 5-HT transport to the inhibitory effects of various psychotropic drugs. Furthermore, we demonstrated the presence of a functional 5-HT uptake system, with low substrate affinity, in human feto-placental endothelial cells. These cells on the fetal side of the feto-placental unit are in direct contact with fetal blood and, therefore, take up fetal 5-HT. Finally, we demonstrated that human fetal platelets express a functional high-affinity 5-HT uptake system similar to that found in adult platelets.

The identification of multiple membrane transport systems for the uptake of extracellular 5-HT at the human maternal–fetal interface (Figure 7) extends our understanding of the mechanisms controlling 5-HT homeostasis during human fetal development. Since cellular 5-HT uptake plays a central role in regulating the local concentrations of 5-HT near its molecular targets, these results may open new avenues for modulating various disorders associated with developmental changes in 5-HT signaling.

## 4. Materials and Methods

### 4.1. Materials

Tritium-labeled serotonin creatinine sulfate (^3^H-serotonin; 28.3 Ci mmol^−1^ and 41.3 Ci mmol^−1^) and radiocarbon-labeled serotonin binoxalate (^14^C-serotonin; 54.0 mCi mmol^−1^) were obtained from Perkin Elmer (Waltham, MA, USA). Serotonin creatinine sulfate, imipramine hydrochloride, citalopram hydrobromide, 3,4-methylenedioxymethamphetamine hydrochloride (MDMA, “Ecstasy”), L-ascorbic acid, and Hanks’ Balanced Salts Solution (HBSS) were obtained from Sigma-Aldrich (St. Louis, MO, USA). Pargyline hydrochloride was from Cayman Chemical (Ann Arbor, MI, USA). Other drugs were generous gifts from manufacturers.

### 4.2. Isolation and Culture of Human Primary Placental Cells

The study was performed in accordance with the protocol approved by Medical University of Graz, Graz, Austria. Written informed consent was obtained from all women who donated placentas for cell isolation. All placentas were from singleton term (37–42 weeks of gestation) pregnancies. The exclusion criteria were pregnancy complications except mild gestational diabetes mellitus and any known fetal or neonatal anomalies. Additional information about the participants can be found in Table S3.

Trophoblast cells were isolated from microvillous tissue as described [71]. Briefly, rinsed placental villous tissue was digested with a mixture of trypsin-dispase-DNAse I (Sigma Aldrich, St. Louis, MO, USA), and the released cells were captured by Percoll-gradient centrifugation. After centrifugation, the cells were purified with immunomagnetic beads conjugated to a murine monoclonal antibody recognizing HLA-class-I antigen (Dynabeads™, Thermo Fisher Scientific, Waltham, MA, USA). Feto-placental endothelial cells were isolated from dissected feto-placental vessels following a standardized protocol as described [72]. In brief, feto-placental vessels dissected from the apical surface of the chorionic plate were perfused for 7 min with HBSS containing collagenase, dispase II (both from Sigma Aldrich, St. Louis, MO, USA), and antibiotics (Thermo Fisher Scientific, Waltham, MA, USA), after which the released cells were collected and washed. Representative cell isolations were tested for identity and purity by immunocytochemical staining of specific cell markers [71,72].

The cells were cultured on gelatin (1%)-coated plates (Corning Inc., Corning, NY, USA), trophoblasts in Dulbecco’s Modified Eagle’s Medium (DMEM; Gibco, Paisley, UK), and feto-placental endothelial cells in Endothelial Cell Basal Medium (EBM; Lonza, Verviers, Belgium) or Endothelial Cell Growth Medium MV (PromoCell, Heidelberg, Germany), each supplemented with 10% fetal bovine serum (FBS; Gibco, Paisley, UK) and 1% antibiotic (Thermo Fisher Scientific, Waltham, MA, USA). Cultures were maintained at 37 °C, 5% CO_2_, and 21% O_2_.

### 4.3. 5-HT Uptake in Human Primary Placental Cells

Cells were seeded onto 24-well plates and left until confluence was reached (usually 1 to 2 days). After removing the medium and rinsing the cells with HBSS, uptake was initiated by adding pre-warmed (37 °C) or pre-cooled (4 °C) uptake buffer (0.2 mL/well). The uptake buffer was HBSS containing a mixture of ^3^H-labeled and unlabeled 5-HT at the required final concentration of 5-HT, as well as ascorbic acid (100 μM) and pargyline (10 μM) to prevent 5-HT oxidation and enzymatic degradation, respectively. Reactions were carried out at 37 °C or on ice for a specified amount of time and were terminated by removing the uptake buffer and adding ice-cold HBSS (1 mL/well). The cells were then washed thoroughly with ice-cold HBSS and lysed in 0.3 M NaOH (0.4 mL/well). Radioactivity in the cell lysates was quantified on Tri-Carb 2100TR Liquid Scintillation Counter, using the Ultima Gold liquid scintillation cocktail (both from Perkin Elmer, Waltham, MA, USA). The total protein concentration in the cell lysates was determined using the Qubit™ Protein Assay Kit (Thermo Fisher Scientific, Waltham, MA, USA). The specific (carrier-mediated) uptake was calculated as the difference between the total uptake (at 37 °C) and non-specific uptake (on ice). All assays were performed in triplicate.

In the initial uptake rate studies, the uptake of 5-HT was measured within an experimentally determined linear time range, at six increasing 5-HT concentrations covering a range typical of a high-affinity (0.1, 0.2, 0.4, 0.8, 1.6, and 3.2 μM) or low-affinity (94, 188, 375, 750, 1500, and 3000 μM) uptake mechanisms. The initial rates of specific 5-HT uptake were fitted to the Michaelis–Menten kinetics model using GraphPad Prism software version 8 (GraphPad Software, LLC, San Diego, CA, USA). Best-fit values of the Michaelis affinity constant (*Km*) and maximal transport velocity (*Vmax*) were estimated by nonlinear least-squares regression analysis.

### 4.4. Pharmacological Studies

A stock solution of MDMA (10^−1^ M) was prepared in DMSO (Sigma Aldrich, St. Louis, MO, USA) and for all other drugs (10^−2^ M) in HBSS. After removing the medium and rinsing with HBSS, the cells were incubated for 10 min in the presence of drug or vehicle (control) and then for 10 min in the presence of ^3^H-5-HT and drug or vehicle. Transport on ice was subtracted from that at 37 °C and the specific uptake in the presence of each drug was expressed as a percentage of the control (vehicle without drug). There were no differences in the 5-HT uptake between the controls prepared with DMSO and HBSS at concentrations equivalent to those in the samples with the drugs. Half-maximal inhibitory concentration (IC_50_) values with 95% confidence intervals (CI) were determined with GraphPad Prism software using nonlinear least-squares regression analysis.

### 4.5. Gene Expression Analyses

Cells for the gene expression analyses were seeded on six-well plates and harvested at confluence. Placental tissue, which served as a positive control, was obtained as described previously [70]. The total RNA was extracted using the RNeasy Plus Mini Kit (Qiagen, Hilden, Germany) according to the manufacturer’s protocol with an optional on-column DNA digestion step. The concentration and purity of RNAs was determined spectrophotometrically (NanoDrop, Witec AG, Littau, Germany). The RNA integrity was assessed using 1% agarose gel electrophoresis. cDNA was synthesized from equal amounts of RNA, using iScript cDNA Synthesis Kit (Bio-Rad, Hercules, CA, USA) according to the manufacturer’s protocol. Control reactions without reverse transcriptase (RT-) were prepared to check for contamination with genomic DNA.

The sequences of the primers (Metabion, Planegg, Germany) used in qualitative end-point PCR and quantitative real-time PCR (qPCR) are listed in Supplementary Table S4 [36,37,42,43,73,74,75,76,77]. Qualitative end-point PCR assays were performed using AllTaq Master Mix (Qiagen, Hilden, Germany) according to the manufacturer’s recommendations. Amplicons obtained at 40 PCR cycles were separated on 2% agarose gels and visualized using Midori Green Advance DNA Stain (Nippon Genetics, Düren, Germany).

qPCR assays were performed on the 7300 Real Time PCR System using Sybr Green Master Mix (both from Applied Biosystems, Waltham, MA, USA), according to the manufacturer’s recommendations. qPCRs of *ACTB* were prepared with 10 ng cDNA and of other genes with 20 ng cDNA per reaction. Assays were run in duplicate or triplicate. The specificity of the qPCR amplicons was verified by agarose gel electrophoresis and melting curve analysis. The qPCR efficiencies of the genes of interest were similar to those of the reference gene *ACTB* (the slope of the log input amount against ΔCq < 0.1), and thereby the relative expression levels were calculated using the comparative Cq (ΔΔCq) method [78].

### 4.6. Isolation of Platelet Rich Plasma from Cord Blood

The study protocol was approved by the Ethics Committee of the Clinical Hospital Centre Zagreb and Bioethics Committee of the Ruđer Bošković Institute, Zagreb, Croatia. All methods were performed in accordance with the relevant guidelines and regulations. Cord blood samples used for platelet isolation were from different pregnancies than those from which the primary placental cells were obtained. Samples were collected from neonates born by elective Cesarean section (Table S2) and whose mothers provided signed informed consent.

The cord blood samples were collected via umbilical vein puncture after the birth of the baby and before the delivery of the placenta and immediately mixed in ACD-A tubes (Greiner Bio-One, Kremsmunster, Austria) with anticoagulant acid citrate dextrose (ACD; Sigma Aldrich, St. Louis, MO, USA) in a ratio of 1:5 (ACD: blood). Platelet rich plasma (PRP) was isolated by centrifugation of anticoagulated blood for 2 min at 1100 g. The total platelet protein content was determined using a Bradford assay. The platelet count and platelet volume were determined in whole blood and PRP samples using the DxH 500 hematology analyzer (Beckman Coulter, Brea, CA, USA). Platelet counts (mean ± SD, n = 9) were 242 ± 112 × 10^6^/mL in whole cord blood and 388 ± 92 × 10^6^/mL in PRP samples. The platelet volumes (mean ± SD, n = 9) in whole cord blood samples (7.8 ± 0.7 fL) and PRP (7.4 ± 0.62 fL) were highly correlated (Spearmen’s correlation coefficient = 0.94, *p* = 0.001, n = 9), indicating that the platelet population isolated in PRP well represents the platelets in cord blood samples.

### 4.7. 5-HT Uptake in Cord Blood Platelets

The uptake of 5-HT in cord blood platelets was measured within two hours after sampling, according to a slightly modified protocol used in our previous studies with adults [47,48]. Briefly, PRP samples (60 μL) were mixed with CaCl_2_-free Krebs–Ringer phosphate buffer (KRB; 840 μL) and pre-incubated in a shaking bath at 37 °C for 10 min. Uptake was initiated by the addition of KRB (100 μL) containing ^14^C-5-HT at final concentrations of 0.15, 0.25, 0.40, 0.70, 1.20, and 2.00 in the reaction mixture. The reactions were carried out at 37 °C for 60 s and terminated by rapid cooling (the addition of ice-cold saline) and immediate vacuum filtration through a glass microfiber filter (Whatman GF/C, GE Healthcare, Chicago, IL, USA). Radioactivity retained on the filters after thorough rinsing was quantified with Tri-Carb 2810TR Liquid Scintillation Analyzer, using the Ultima Gold MV liquid scintillation cocktail (both from Perkin Elmer, Waltham, MA, USA). Non-specific uptake was measured using the same procedure but at approximately 4 °C (ice bath). All assays were performed in duplicate.

The specific (carrier-mediated) uptake, calculated as the difference between the total (at 37 °C) and nonspecific (ice bath) uptake, was expressed per platelet count (for comparison with published data for adult platelets) and per total platelet protein (for comparison with results in human primary trophoblasts and feto-placental endothelial cells). Values of *Km* and *Vmax* were determined as described for primary placental cells.

### 4.8. Statistical Analysis

Statistical analyses were performed using GraphPad Prism version 8 (GraphPad Software, LLC, San Diego, CA, USA). The distribution of data was tested using the D’Agostino–Pearson omnibus normality test. Normally distributed data were analyzed using Student’s *t*-test, one-way analysis of variance (ANOVA), or two-way ANOVA. Non-normally distributed data were analyzed using the Mann–Whitney test. The statistical tests applied are specified in the figure legends. The significance level was set at 0.05.

## Data Availability

The data generated for this study are available from the corresponding author on reasonable request.

## Supplementary Materials

The following are available online at https://www.mdpi.com/article/10.3390/ijms22157807/s1, Figure S1: Time course of the specific uptake of 5-HT into human primary trophoblasts and feto-placental endothelial cells, Figure S2: Uptake of 5-HT into feto-placental endothelial cells, measured over the high-affinity range of 5-HT concentrations, Figure S3: RT-qPCR analysis of *SERT*, *PMAT*, *OCT1*, -*2*, and -*3* mRNAs in pooled samples of human primary trophoblasts and feto-placental endothelial cells, Table S1: Kinetic parameters of 5-HT uptake in human primary trophoblasts, feto-placental endothelial cells, and cord blood platelets, Table S2: Quantification cycle values obtained in RT-qPCR analysis of individual trophoblast and feto-placental endothelial cell samples, Table S3: Characteristics of participants who donated tissue for trophoblast, feto-placental endothelial cell, and cord blood platelet isolation for the study, Table S4: The primer sequences used in qualitative end-point and quantitative real-time PCR analyses.
