# Supplementary material for: Differential Serotonin Uptake Mechanisms at the Human Maternal–Fetal Interface
Source: Int J Mol Sci. 2021 Jul 21;22(15):7807. doi: 10.3390/ijms22157807 (PMC8346107; doi:10.3390/ijms22157807)
Supplement: Supplementary file 1 [file ijms-22-07807-s001.zip › ijms-1273248-supplementary.pdf]

SUPPLEMENTARY MATERIAL

Differential serotonin uptake mechanisms at the human maternal-fetal interface

Petra Baković[&], Maja Kesić[&], Maja Perić, Ivona Bečeheli, Marina Horvatiček, Meekha George, Lipa Čičin-Šain, Gernot Desoye, Christian Wadsack, Ute Panzenboeck[#], Jasminka Štefulj[*]

[&]PB and MK contributed equally to this work

[#]deceased

[*]correspondence to: *jasminka.stefulj@irb.hr*

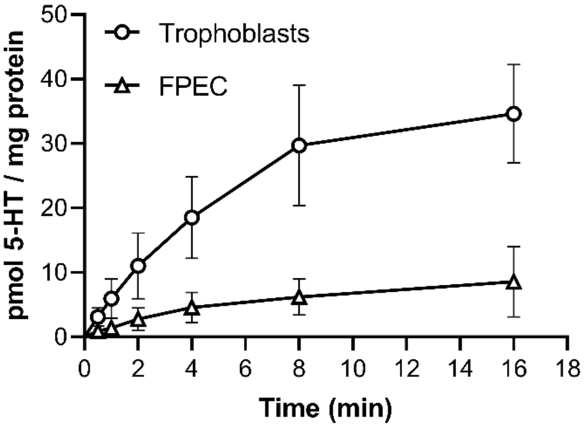

Figure S1. Time course of specific uptake of 5-HT into human primary trophoblasts and feto-placental endothelial cells. Cells were incubated in the presence of ^{3}H-5-HT (10^{-7} M) at 37°C or on ice for the indicated time. Specific uptake, calculated as the difference between transport at 37°C and on ice, is expressed per total cellular protein content. Values are means ± SD from two different donors, each analyzed in triplicate. Under the conditions used (10^{-7} M 5-HT), the specific uptake of 5-HT was significantly higher (up to 5-fold) in trophoblasts than in fetoplacental endothelial cells ($p<0.0001$, 2-way ANOVA).

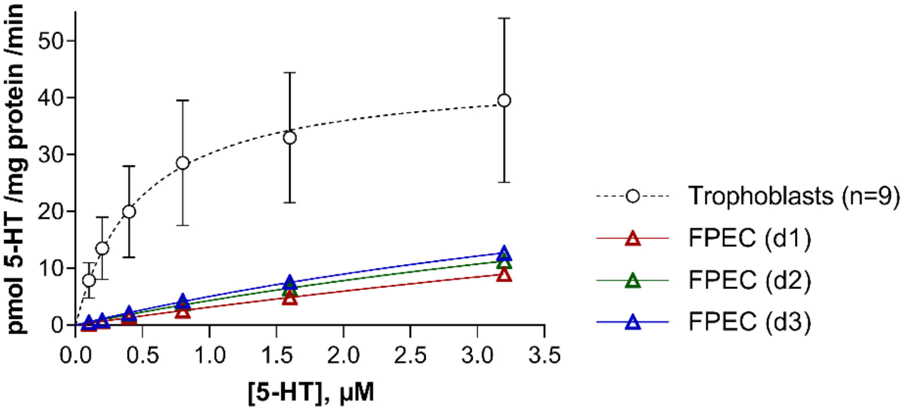

Figure S2. Uptake of 5-HT into feto-placental endothelial cells (FPEC), measured over the high-affinity range of 5-HT concentrations (0.1 to 3.2 µM). Specific transport was calculated as the difference between transport at 37°C and on ice. Shown are FPEC from three different donors (d), each analyzed in triplicates. For comparison, a curve based on the mean values of trophoblasts from 9 different donors (mean ± SEM), each analyzed in triplicates, is included in the graph (dashed line). The rates of specific 5-HT uptake in FPEC were not only greatly reduced compared to trophoblasts, but also did not show clear saturation kinetics within the high-affinity range of 5-HT concentrations, preventing estimation of kinetic parameters with acceptable accuracy and indicating the lack of a functional high-affinity uptake system as observed in trophoblasts.

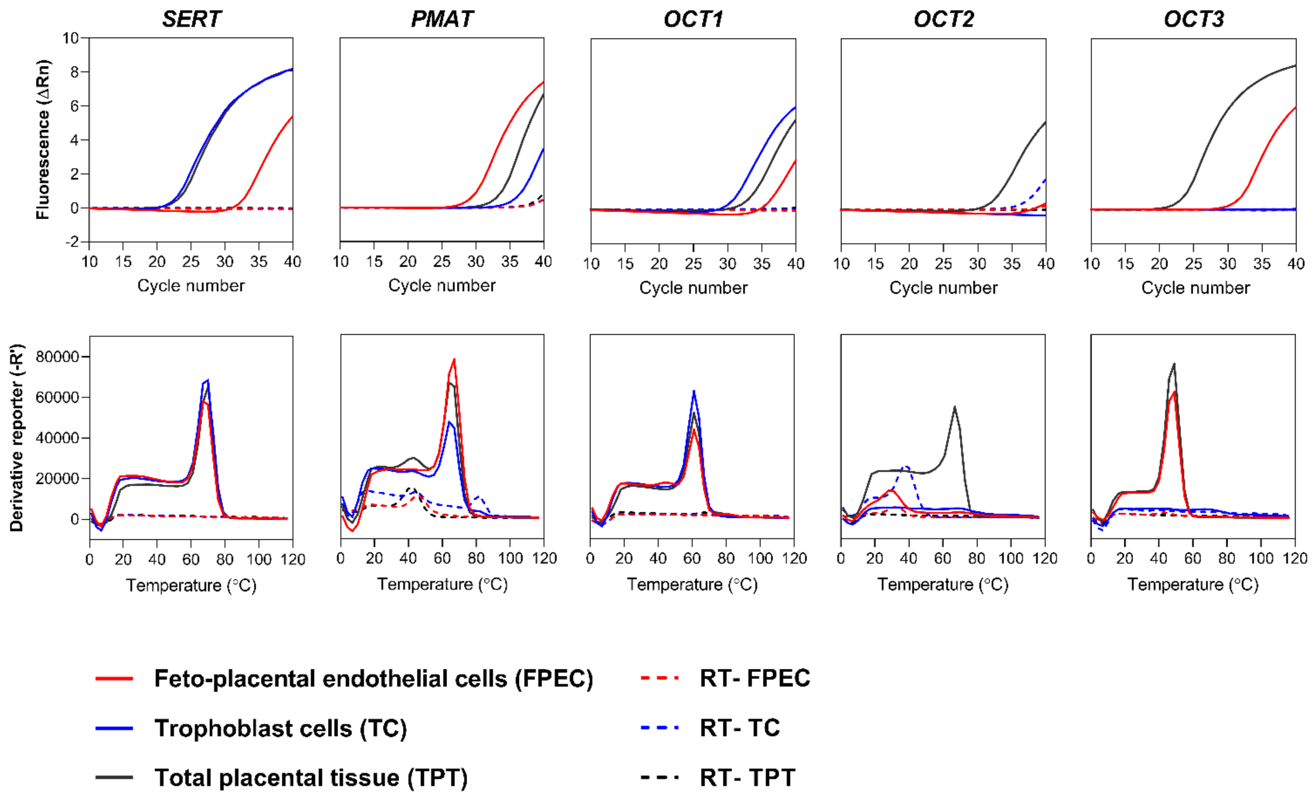

Figure S3. Reverse transcription - real-time quantitative PCR (RT-qPCR) analysis of serotonin transporter (*SERT*), plasma membrane monoamine transporter (*PMAT*), organic cation 1 (*OCT1*), 2 (*OCT2*) and 3 (*OCT3*) mRNAs in pooled samples of human primary trophoblasts (n=9) and feto-placental endothelial cells (n=12). Total placental tissue (a pool of 20 donors) served as a positive control. Amplification curves (top row) and melting curves (bottom row) are shown. Amplification curves show the increase in fluorescence (baseline-corrected normalized reporter; ΔRn) against the number of cycles. Melting curves showing the specificity of the amplified products are plotted as derivative reporter (the negative first-derivative of the normalized fluorescence generated by the reporter; -R′) versus temperature (°C). RT- FPEC, RT- TC, and RT- TPT - control reaction without reverse transcriptase, prepared with RNA from feto-placental endothelial cells, trophoblast cells, and total placental tissue, respectively.

Table S1. Kinetic parameters of 5-HT uptake in human primary trophoblasts, feto-placental endothelial cells, and cord blood platelets. Means ± SD or medians with interquartile range (IQR, in parentheses) are given for data with normal ad non-normal distributions, respectively. Data for trophoblasts and feto-placental endothelial cells are from Figure 2C and for cord blood platelets from Figure 5B. *Km* - Michaelis affinity constant, *Vmax* - maximal transport velocity, n - number of participants analyzed.

**Cells**	**n**	***Km*** (µmol /L)	***Vmax*** (pmol /mg protein /min)
Trophoblasts	9	0.64 ± 0.27	25 (20 - 68)
Feto-placental endothelial cells	9	782 ± 218	1005 ± 251
Cord blood platelets	9	0.65 ± 0.18	878 ± 286

Table S2. Quantification cycle (Cq) values obtained in reverse transcription - quantitative real-time PCR (RT-qPCR) analysis of individual trophoblast and feto-placental endothelial cell samples. n, number of samples analyzed; ✚, number of samples in which a specific signal was detected; n.a., not applicable.

	**Trophoblasts (n = 9)**		**Feto-placental endothelial cells (n = 12)**	
**Gene**	**✚/n**	**Cq, mean (range)**	**✚/n**	**Cq, mean (range)**
SERT	9/9	19.8 (18.2-20.9)	12/12	31.2 (26.8-36.7)
PMAT	9/9	33.0 (31.1-35.1)	12/12	25.0 (23.2-26.5)
OCT1	9/9	31.7 (29.9-33.8)	9/12	35.1 (34.1-36.7)
OCT2	0/9	n.a.	0/12	n.a.
OCT3	3/9	35.5 (34.3-37.1)	5/12	32.2 (28.4-35-1)
MAOA	9/9	21.2 (18.6-23.1)	12/12	23.3 (22.3-24.7)
ACTB	9/9	14.2 (12.5-15.1)	12/12	13.8 (13.2-15.5)

Table S3. Characteristics of participants who donated tissue for trophoblast, feto-placental endothelial cell, and cord blood platelet isolation for the study. Continuous variables are shown as mean ± SD.

Characteristic	Trophoblasts	Feto-placental endothelial cells	Cord blood platelets
Number of participants	16 [a]	21 [b]	9
Labor (vaginal : Cesarean)	5 : 11	10 : 11	0 : 9
Newborn's sex (male : female)	10 : 6	10 : 11	4 : 5
Maternal age (years)	29.4 ± 6.5	31.5 ± 5.2	32.8 ± 5.1
Gestational age (weeks)	39.5 ± 1.2	38.8 ± 1.1	39.1 ± 0.7
Birth weight (g)	3462 ± 447	3228 ± 430	3653 ± 616
Birth length (cm)	50.5 ± 2.3	49.6 ± 1.7	50.7 ± 2.3

[a] Donors included in time-course (n=2), kinetic (n=9), pharmacological (n=8), and mRNA (n=9) experiments partially overlapped.

[b] Donors included in time-course (n=2), kinetic (n=12), citalopram (n=2), and mRNA (n=12) experiments partially overlapped.

Table S4. Sequences of gene-specific primers used in qualitative end-point and quantitative real-time PCR analyses (f - forward, r – reverse).

Gene symbol	Gene name	Primer sequence (5' - 3')	Amplicon (bp)	Source
SERT / SLC6A4	serotonin transporter	f: TGGTTCTATGGCATCACTCAGTTC<br>r: GTTGTGGCGGGCTCATCAG	148 [a,b]	[1]
PMAT / SLC29A4	plasma membrane monoamine transporter	f: TTTTATCAGCATCTGCGACG<br>r: CCCACGAAGTCTGACAGGTT	762 [a]	[2]
PMAT / SLC29A4	plasma membrane monoamine transporter	f: CTGTCCTCCTGAACAACGTCC<br>r: ACACGTCGCAGATGCTGATAA	111 [b]	[3] [c]
OCT1 / SLC22A1	organic cation transporter 1	f: CTGTGTAGACCCCCTGGCTA<br>r: GTGTAGCCAGCCATCCAGTT	363 [a]	[4]
OCT1 / SLC22A1	organic cation transporter 1	f: GACGCCGAGAACCTTGGG<br>r: GGGTAGGCAAGTATGAGG	198 [b]	[5]
OCT2 / SLC22A2	organic cation transporter 2	f: CCTGGTATGTGCCAACTCCT<br>r: CACCAGGAGCCCAACTGTAT	334 [a]	[4]
OCT2 / SLC22A2	organic cation transporter 2	f: CGGAGATATCGGAGAACAGT<br>r: GCATTCTTATTCTGGGAGATC	200 [b]	[5]
OCT3 / SLC22A3	organic cation transporter 3	f: CAGAGATCACTGTTACAGAT<br>r: GATAGCTCCTTCTTTCTGTC	734 [a]	[6]
OCT3 / SLC22A3	organic cation transporter 3	f: GAGGACCACAGTGGCTACAT<br>r: ACAGACCTGAACAGAGCGAA	128 [b]	[7]
MAOA	monoamine oxidase A	f: GAGCGGCTACATGGAAGGG<br>r: TCACCTTCCCGAGACCATTTA	77 [a,b]	[8]
ACTB	actin beta	f: TCCCTGGAGAAGAGCTACG<br>r: GTAGTTTCGTGGATGCCACA	131 [a,b]	[9]

[a] primers used in conventional end-point PCR analysis

[b] primers used in real-time PCR analysis

[c] PrimerBank ID: 100913033c2

Supplementary References

1. Van Lelyveld, N.; Ter Linde, J.; Schipper, M.E.I.; Samsom, M. Regional differences in expression of TPH-1, SERT, 5-HT3 and 5-HT4 receptors in the human stomach and duodenum. *Neurogastroenterol. Motil.* **2007**, *19*, 342–348

2. Naganuma, F.; Yoshikawa, T.; Nakamura, T.; Iida, T.; Harada, R.; Mohsen, A.S.; Miura, Y.; Yanai, K. Predominant role of plasma membrane monoamine transporters in monoamine transport in 1321N1, a

human astrocytoma-derived cell line. *J. Neurochem.* **2014**, *129*, 591–601.

3. Spandidos, A.; Wang, X.; Wang, H.; Seed, B. PrimerBank: A resource of human and mouse PCR primer pairs for gene expression detection and quantification. *Nucleic Acids Res.* **2009**, *38*, 792–799.

4. Li, R.W.S.; Yang, C.; Kwan, Y.W.; Chan, S.W.; Lee, S.M.Y.; Leung, G.P.H.; Luo, J.-D. Involvement of organic cation transporter-3 and plasma membrane monoamine transporter in serotonin uptake in human brain vascular smooth muscle cells. **2013**, *4* .

5. Hayer-Zillgen, M.; Brüss, M.; Bönisch, H. Expression and pharmacological profile of the human organic cation transporters hOCT1, hOCT2 and hOCT3. *Br J Pharmacol*. **2002**, *136(6)*, 829-836.

6. Sata, R.; Ohtani, H.; Tsujimoto, M.; Murakami, H.; Koyabu, N.; Nakamura, T.; Uchiumi, T.; Kuwano, M.; Nagata, H.; Tsukimori, K.; Nakano, H.; Sawada, Y. Functional analysis of organic cation transporter 3 expressed in human placenta. *J Pharmacol Exp Ther*. **2005**, *315(2)*, 888-895.

7. Real-time PCR (TaqMan) primer and probes design tool. Available online: https://www.genscript.com/tools/real-time-pcr-taqman-primer-design-tool (accessed on 11.3.2021.).

8. Sun, Y.; Zhang, J.; Yuan, Y.; Yu, X.; Shen, Y.; Xu, Q. Study of a possible role of the monoamine oxidase A (MAOA) gene in paranoid schizophrenia among a Chinese population. *Am. J. Med. Genet. Part B Neuropsychiatr. Genet.* **2012**, *159 B*, 104–111.

9. Métayé, T.; Menet, E.; Guilhot, J.; Kraimps, J.-L. Expression and Activity of G Protein-Coupled Receptor Kinases in Differentiated Thyroid Carcinoma. *J. Clin. Endocrinol. Metab.* **2002**, *87*, 3279–3286.
